# Collider Bias Is an Insufficient Explanation for the Inverse Obesity Paradox in Prostate Cancer

**DOI:** 10.1002/cam4.70871

**Published:** 2025-04-15

**Authors:** Tanja Stocks, Christel Häggström, Josef Fritz

**Affiliations:** ^1^ Department of Translational Medicine Lund University Malmö Sweden; ^2^ Northern Registry Centre, Department of Diagnostics and Intervention, Oncology Umeå University Umeå Sweden; ^3^ Institute of Clinical Epidemiology, Public Health, Health Economics, Medical Statistics and Informatics Medical University of Innsbruck Innsbruck Austria

**Keywords:** body mass index, case‐only analysis, collider bias, obesity paradox, prostate cancer, simulation

## Abstract

**Background:**

Collider bias is often considered a potential explanation when the association between obesity and disease diagnosis differs from that with disease outcome, as seen in the “obesity paradox.” For prostate cancer (PCa), in particular localized PCa, an “inverse” obesity paradox has been observed, where body mass index (BMI) is negatively associated with diagnosis (hazard ratio [HR] ~0.9 per 5‐kg/m^2^ increase), but positively associated with PCa‐specific death (HR ~ 1.2). However, collider bias in this context remains unexplored.

**Methods:**

We simulated binary disease diagnosis and outcome data, including the typically unmeasured/unknown background variable (U) that could introduce collider bias. We calculated U‐unadjusted (biased) and U‐adjusted (true) marginal odds ratios (OR) from a case‐only analysis, and determined the bias percentage using $\left({\text{OR}}_{\text{Biased}}-{\text{OR}}_{\text{True}}\right)/{\text{OR}}_{\text{True}}\times 100$. Similar simulations were performed for classical confounding.

**Results:**

Across a broad range of plausible parameter values for the PCa context, collider bias did not distort the OR of BMI on PCa death by more than 4%, equivalent to a ± 0.04 distortion in the OR estimate for continuous BMI. In comparison, classical confounding showed a higher potential for distorting BMI and PCa death associations than collider bias.

**Conclusions:**

Collider bias alone is unlikely to explain the inverse obesity paradox in (localized) PCa, reinforcing some mechanistic evidence that the observed positive relationship between BMI and PCa death is real, and not a statistical artifact. This finding emphasizes the importance of exploring alternative mechanisms beyond collider bias to better understand the underlying factors driving this paradox.

## 1 Introduction

**FIGURE 1:**
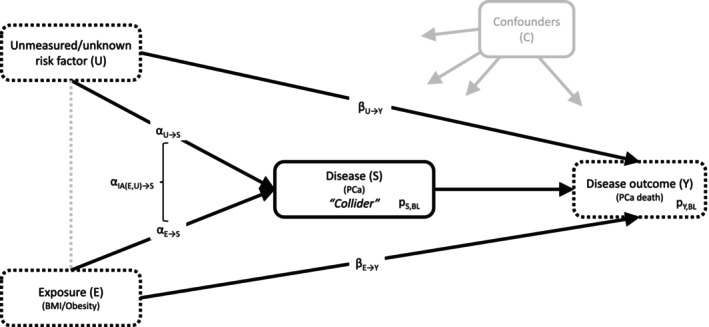
Collider stratification bias scenario depicted as a causal diagram (directed acyclic graph, DAG): An exposure (E) and an unmeasured/unknown risk factor (U) causally affect the risk of the disease of interest (S; the so called collider). Both E and U also causally affect a specific disease outcome (Y). U might represent a single risk factor or the combined effects of multiple risk factors operating in concert, for example, genetic risk, family history, environmental factors etc. Even though E and U are not causally related and thus U does not confound the association between E and Y in the full population (i.e., both disease‐free and diseased individuals), it might confound the E‐Y association in an analysis of diseased individuals only, because the restriction to cases only (i.e., collider stratification) creates a spurious association between E and U (indicated via the gray dotted line). In theory, adjusting case‐only analyses for U would prevent collider bias from occurring, but in practice this is impossible because U is unmeasured or even unknown. In our simulation analyses, we consider both binary and continuous variables E and U, and binary variables S and Y, and we assume that E and U increase the log odds of S and Y by the values α_E→S_, α_U→S_, β_E→Y_, and β_U→Y_. We also allow for interaction between E and U on the odds of S through the interaction term α_IA(E,U)_→S. Together with the baseline probabilities p_S,BL_ of being diseased and p_Y,BL_ of the disease outcome occurring, these form the input parameters of our simulations. For the sake of readability, we do not display any potential confounders C between variables E, U, S, and Y, but implicitly assume throughout the whole text that all analyses are also correctly adjusted for C. BMI, body mass index; PCa, prostate cancer.

Obesity is associated with a lower risk of diagnosis of low‐risk, commonly screen‐detected prostate cancer (PCa), but is unassociated or even slightly positively associated with the risk of more advanced PCa [1, 2, 3]. In contrast, obesity has consistently been associated with worse PCa‐specific survival, both in prospective cohort studies following the whole, initially cancer‐free cohort from the time of study inclusion and in PCa case‐only studies [2, 3, 4, 5, 6]. The results from the full cohort analyses reflect the association of obesity with a mix of time from study entry until PCa diagnosis and from PCa diagnosis to PCa‐specific death. The outcome of primary interest, time from PCa diagnosis to PCa death, that is, survival from PCa, is more directly studied in analyses of PCa cases only. However, the inclusion of PCa cases only in these analyses might introduce collider stratification bias (short: collider bias), a special kind of selection bias inducing a noncausal association between exposure and outcome in the selected data. The causal diagram in Figure 1 illustrates the underlying conceptual framework and assumptions for the PCa scenario. Obesity affects PCa risk (the collider), and there are unmeasured and/or unknown variables that affect both PCa risk and death (e.g., genetic factors). The presence of these two associations is sufficient to introduce collider bias.

The potential impact of collider bias on analyses of cancer cases only has been well studied in the context of the “obesity paradox”, referring to settings where obesity is associated with an increased disease risk, but better disease survival (observed for e.g., lymphoma, leukemia, colorectal, endometrial, thyroid, and renal cancers) [7, 8, 9, 10, 11]. For localized PCa, men with obesity have a decreased risk of diagnosis, but worse survival, which can be seen as an “inverse” form of the obesity paradox. Since the introduction of prostate‐specific antigen (PSA) testing for early detection of PCa, localized PCa has made up the majority of PCa's detected in developed countries [12, 13]. However, the potential impact of collider bias on the survival of patients with localized PCa has not been investigated. However, risk factors for PCa development and progression, such as genetic factors, which are usually not adjusted for in epidemiological research, have the potential to introduce such bias. In this study, we used simulation analyses to quantify the likely magnitude of collider bias for the association between obesity and PCa death observed in analyses of localized PCa cases only.

## 2 Methods

We generated simulated data of variables E (exposure; either normally distributed or binary), S (disease indicator; binary), Y (survival outcome; binary), and U (unmeasured/unknown risk factor for disease and survival; either normally distributed or binary), as depicted in Figure 1 [2].

Our simulation input parameters were based on previous results from Swedish cohort data [2], where 3.9% of the whole study population (*N*~370,000; mean baseline age 37.5 years) developed a localized “low‐ or intermediate‐risk” PCa (defined as T1‐T2, Gleason score 2–7, PSA < 20 ng/mL, N0/NX, and M0/MX) during on average 28 years of follow‐up, with a mean (standard deviation [SD]) age at diagnosis of 67.2 (7.4) years. Within the group of localized PCa cases, 4.1% subsequently died because of the cancer. A 5‐kg/m^2^ increase in body mass index (BMI) was associated with an 11% decreased risk of localized PCa (hazard ratio [HR] = 0.89; translating to a HR of 0.92 per 1‐SD BMI increase), whereas it was associated with an 18% increased risk of PCa death (HR = 1.18; HR_per 1‐SD BMI increase_ = 1.12), after stratification on cohort and birth decade, and adjustment for age, smoking status, healthcare region, country of birth, highest attained education level, and also for income, marital status, Charlson comorbidity index, and PCa treatment in case‐only analyses.

For our simulations, we used the following data‐generating model:

- E and U independent,
- $\mathrm{Pr}\;\left(S=1\right)=\frac{\exp\left(\alpha_{0}+\alpha_{E\to S}*E+\alpha_{U\to S}*U+\alpha_{\mathrm{IA}\left(E,U\right)\to S}*E*U\right)}{1+\exp\left(\alpha_{0}+\alpha_{E\to S}*E+\alpha_{U\to S}*U+\alpha_{\mathrm{IA}\left(E,U\right)\to S}*E*U\right)}$, and
- $\mathrm{Pr}\;\left(Y=1|S=1\right)=\frac{\exp\left(\beta_{0}+\beta_{E\to Y}*E+\beta_{U\to Y}*U\right)}{1+\exp\left(\beta_{0}+\beta_{E\to Y}*E+\beta_{U\to Y}*U\right)}$.

Thus, $\alpha_{E\to S}$, $\alpha_{U\to S}$, $\alpha_{\mathrm{IA}\left(E,U\right)\to S}$, $\beta_{E\to Y}$, and $\beta_{U\to Y}$ can be interpreted as logarithmized odds ratios (ORs). These were five of the seven input parameters of our simulations. We allowed for interaction between E and U on the odds of S because prior work has shown that the magnitude of the collider bias is substantially larger when such an interaction is present [14, 15]. The other two parameters, $\alpha_{0}$ and $\beta_{0}$, were implicitly determined by specifying the baseline probabilities of *S* = 1 and *Y* = 1 within the group *S* = 1. The sample size was chosen to be 500,000. As outcomes are rare (~4% each), the approximation of HRs derived from time‐to‐event data by ORs derived from binary data is justified.

After creating the simulated dataset, we calculated beta estimates for associations between E, U, S, and Y from the regression models (logistic if the dependent variable was binary, linear otherwise). The magnitude of collider bias was quantified by the percentage of bias (“percentage bias”) using the formula $\left({\text{OR}}_{\text{Biased}}-{\text{OR}}_{\text{True}}\right)/{\text{OR}}_{\text{True}}\times 100$, where ${\text{OR}}_{\text{Biased}}$ is the unadjusted (“collider‐biased”), and ${\text{OR}}_{\text{True}}$ the (for U) adjusted (“unbiased”) marginal OR of E on Y within the subset *S* = 1. ${\text{OR}}_{\text{True}}$, a marginal OR from analyses adjusted for U, was derived as outlined by Zhang [16]. A generic definition of ${\text{OR}}_{\text{True}}$ using counterfactuals and further details were provided in Daniel et al. [17]. Marginalization is essential for a valid comparison of unadjusted versus adjusted ORs because of the non‐collapsibility property of the OR [17].

Additionally, to compare collider versus confounder bias, we generated data simulating a classical confounder U (normally distributed) of the relationship between E (normally distributed or binary) and Y (binary), compared unadjusted (“confounder‐biased”) and (for U) adjusted (“unbiased”) marginal ORs of E on Y, and calculated the percentage bias as outlined above, but applied to confounder‐induced bias. We used the following data‐generating model:

- $\mathrm{Pr}\;\left(E=1\right)=\frac{\exp\left(\gamma_{0}+\gamma_{U\to E}*U\right)}{1+\exp\left(\gamma_{0}+\gamma_{U\to E}*U\right)}$ for E binary; and $\left(E,U\right)\sim N_{2}\left(\left[\begin{array}{c} 0\\ 0 \end{array}\right],\left[\begin{array}{cc} 1 & \rho \\ \rho & 1 \end{array}\right]\right)$ for E continuous, where *N*_2_ denotes a bivariate normal distribution, and ρ is the Pearson correlation coefficient between E and U, and
- $\mathrm{Pr}\;\left(Y=1\right)=\frac{\exp\left(\theta_{0}+\theta_{E\to Y}*E+\theta_{U\to Y}*U\right)}{1+\exp\left(\theta_{0}+\theta_{E\to Y}*E+\theta_{U\to Y}*U\right)}$.

Simulations were conducted using R, version 4.0.5 (R Foundation) [18]. The analysis code is provided as Appendix S1.

## 3 Results

Scenarios 1–10 in Table 1 show the simulation results of plausible scenarios in the context of BMI and PCa [2]. In all scenarios, including those assuming extremely strong effects of U (scenarios 3 and 4) and those incorporating E–U interaction on *S* (scenarios 5–7), the difference between collider‐biased and unbiased ORs of E on Y within *S* = 1 was small (generally ≤ 0.04), as was the percentage bias (in general < 4%). The bias was of similar size for continuous (scenarios 1 and 2) and binary (scenarios 8–10) exposures E.

**TABLE 1:** Results of the simulations showing the magnitude of collider stratification bias for various combinations of input parameters.

Description	Sce‐nario	Simulation settings/input parameters									Simulation results				
											Marginalized odds ratios between E and Y in the subset with *S* = 1			Corr. (ρ) between E and U…^f^	
		Distributions^a^		Probabilities		Odds ratios					Collider‐biased OR^b^, ^c^	Un‐biased OR^b^, ^d^	Percentage bias^e^	In whole sample^b^	Within *S* = 1^b^
		E	U	*p*_S,BL_	*p*_Y,BL_	E→S	E→Y	U→S	U→Y	IA_E,U_→Y					
Plausible BMI—PCa scenarios															
Using continuous E^g^	1	*N* (0, 1)	*N* (0, 1)	0.04	0.04	0.92	1.12	3	3	No	1.124	1.116	0.8% (0.7%)	< 0.001	0.007
	2		B (1, 0.25)					5	5	No	1.127	1.124	0.3% (0.4%)	0.002	0.005
	3		*N* (0, 1)					10	10	No	1.121	1.104	1.6% (0.7%)	< 0.001	0.016
	4		B (1, 0.25)					25	25	No	1.124	1.121	0.2% (0.3%)	0.001	0.006
	5		*N* (0, 1)					3	3	1.04	1.150	1.114	3.2% (0.7%)	−0.003	0.034
	6		*N* (0, 1)					3	3	0.96	1.107	1.119	−1.1% (0.7%)	−0.002	−0.02
	7		*N* (0, 1)					10	10	1.04	1.139	1.103	3.2% (0.5%)	0.001	0.02
Using binary E^g^, ^h^	8	*B* (1, 0.10)	*N* (0, 1)	0.04	0.04	0.65	1.63	3	3	No	1.588	1.567	1.4% (2.7%)	< 0.001	0.005
	9		B (1, 0.25)					5	5	No	1.630	1.617	0.8% (1.7%)	0.001	0.004
	10		N (0, 1)					3	3	1.10	1.724	1.599	7.8% (2.6%)	−0.001	0.023
Hypothetical scenarios															
Modified effect of E on S from scenario 1	11	*N* (0, 1)	*N* (0, 1)	0.04	0.04	0.67	1.12	3	3	No	1.170	1.114	5.1% (0.9%)	−0.002	0.038
	12					0.33					1.275	1.112	14.7% (1.1%)	−0.002	0.117
Strong interaction between E and U on Y	13	*N* (0, 1)	*N* (0, 1)	0.04	0.04	0.92	1.12	3	3	2	1.407	1.121	25.6% (1.5%)	< 0.001	0.400
	14	*N* (0, 1)	*N* (0, 1)	0.04	0.04	0.92	1.12	3	3	0.5	0.886	1.120	−20.8% (0.9%)	< 0.001	−0.356
	15	*N* (0, 1)	*N* (0, 1)	0.04	0.04	0.92	1.12	1	3	0.5	0.651	1.116	−41.6% (1.1%)	< 0.001	−0.568
Modified probabilites of S and Y from scenario 1	16	*N* (0, 1)	*N* (0, 1)	0.5	0.04	0.92	1.12	3	3	No	1.130	1.115	1.3% (0.1%)	−0.001	0.015
	17				0.5						1.116	1.098	1.6% (0.1%)	< 0.001	0.019
Modified effect of E on Y from scenario 1	18	*N* (0, 1)	*N* (0, 1)	0.04	0.04	0.92	2	3	3	No	1.926	1.940	−0.7% (0.7%)	< 0.001	0.007
Several modified input parameters	19	*N* (0, 1)	*N* (0, 1)	0.5	0.5	0.92	1.04	3	3	No	1.045	1.033	1.2% (0.1%)	−0.003	0.014
	20			0.04	0.04	0.33	1.12	0.33		No	1.009	1.113	−9.4% (0.9%)	−0.002	−0.120

*Note:* Each scenario was simulated 100 times with a sample size of 500,000 each.

Abbreviations: BMI, body mass index; IA, interaction; OR, odds ratio; PCa, prostate cancer.

^a^ *N* (0, 1)—normally distributed with mean 0 and variance 1; *B* (1, *p*)—Bernoulli distributed with probability *p* for the value 1 and 1‐*p* for the value 0.

^b^ Mean from the 100 simulations.

^c^ Unadjusted for U.

^d^ Adjusted for U.

^e^ Mean (standard deviation) from the 100 simulations. The percentage bias was calculated as $\left({\text{OR}}_{\text{Biased}}-{\text{OR}}_{\text{True}}\right)/{\text{OR}}_{\text{True}}\times 100$, where ${\text{OR}}_{\text{Biased}}$ is the unadjusted (“collider‐biased”), and ${\text{OR}}_{\text{True}}$ the (for U) adjusted (“unbiased”) marginal OR of E on Y.

^f^ Given as Pearson correlation coefficients ρ.

^g^ By selecting different ORs for normally distributed and binary U's, we account for the fact that an OR is raised to the power of $\sqrt{2}$ after dichotomization [19], thus mimicking similar effects sizes.

^h^ A prevalence of 10% for a binary exposure E, OR of E on S of 0.65, and OR of E on Y of 1.63 mimic the numbers in the Swedish cohorts [2] for obesity versus normal weight.

**FIGURE 2:**
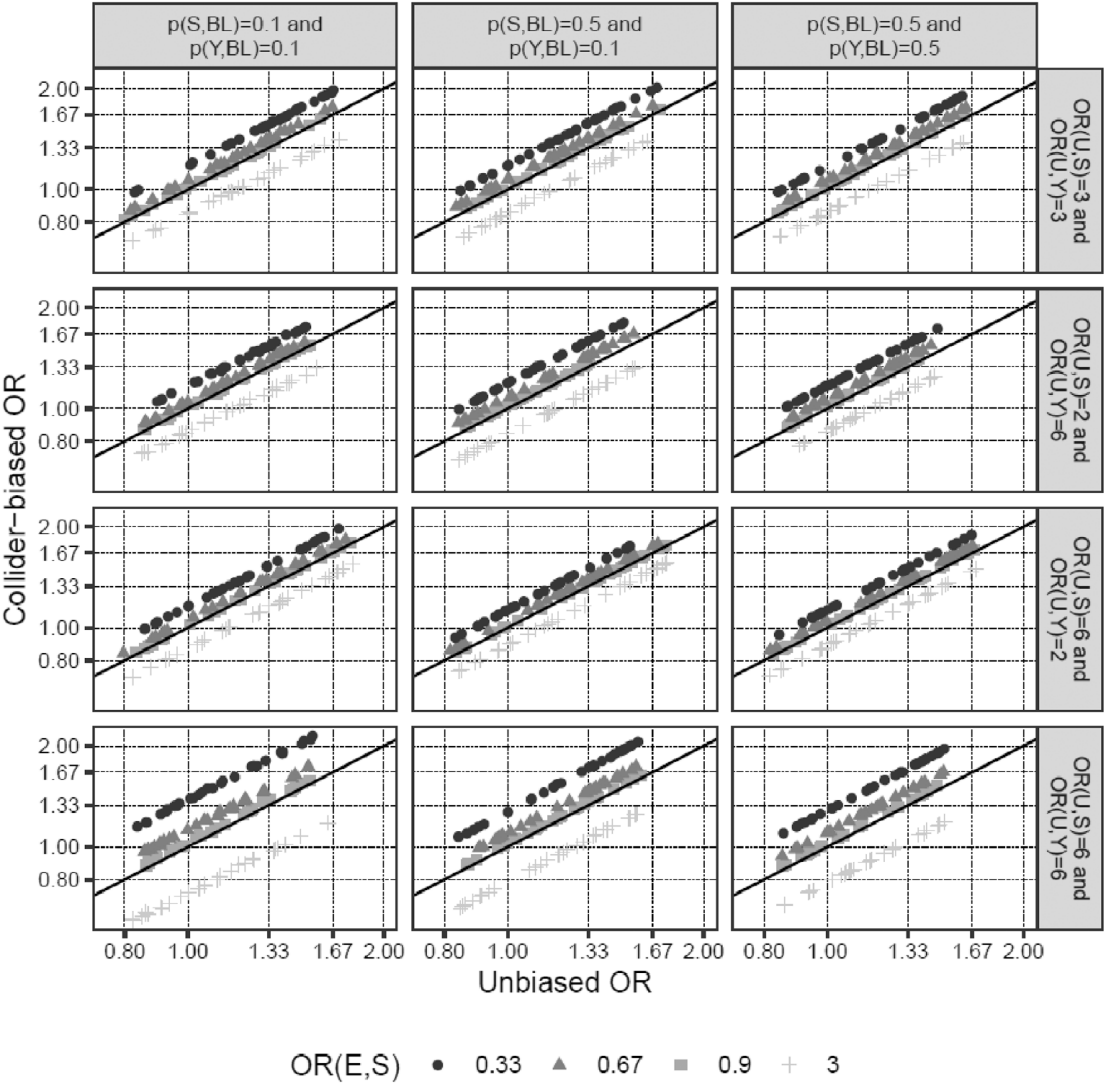
Unbiased (x‐axis) versus collider‐biased (y‐axis) marginal ORs of E on Y for different combinations of input parameters in the absence of an interaction between E and U on S on the log(OR)‐scale. The three columns show the impact of disease (*p*_S,BL_) and disease outcome (p_Y,BL_) baseline probabilities (values of 0.1 and 0.5, respectively) on the magnitude of bias, and the four rows the impact of the strength of the associations of U with S and Y (OR_U→S_ and OR_U→Y_ varying between 2 and 6). One dot/mark corresponds to one simulation based on a sample size of 500,000. As long as OR_E→S_ is small (i.e., 0.9), the differences between unbiased and collider‐biased ORs remain small as well, even in cases of strong effects of U on both S and Y (bottom row). For the magnitude of collider bias to be substantial, all three effects OR_E→S_, OR_U→S_, and OR_U→Y_ must be strong; if one of the three effects is weak the collider bias is immediately attenuated. The specific baseline probabilities of the disease (*p*_S,BL_) and disease outcome (*p*_Y,BL_) (different scenarios were simulated column‐wise) have hardly any influence on the magnitude of the collider bias. As seen from simulation results lining up on parallel lines for different scenarios, the ratio of the collider‐biased vs. unbiased ORs of E on Y within the subset *S* = 1 is invariant to the actual value of the OR_E→Y_. OR, odds ratio.

**FIGURE 3:**
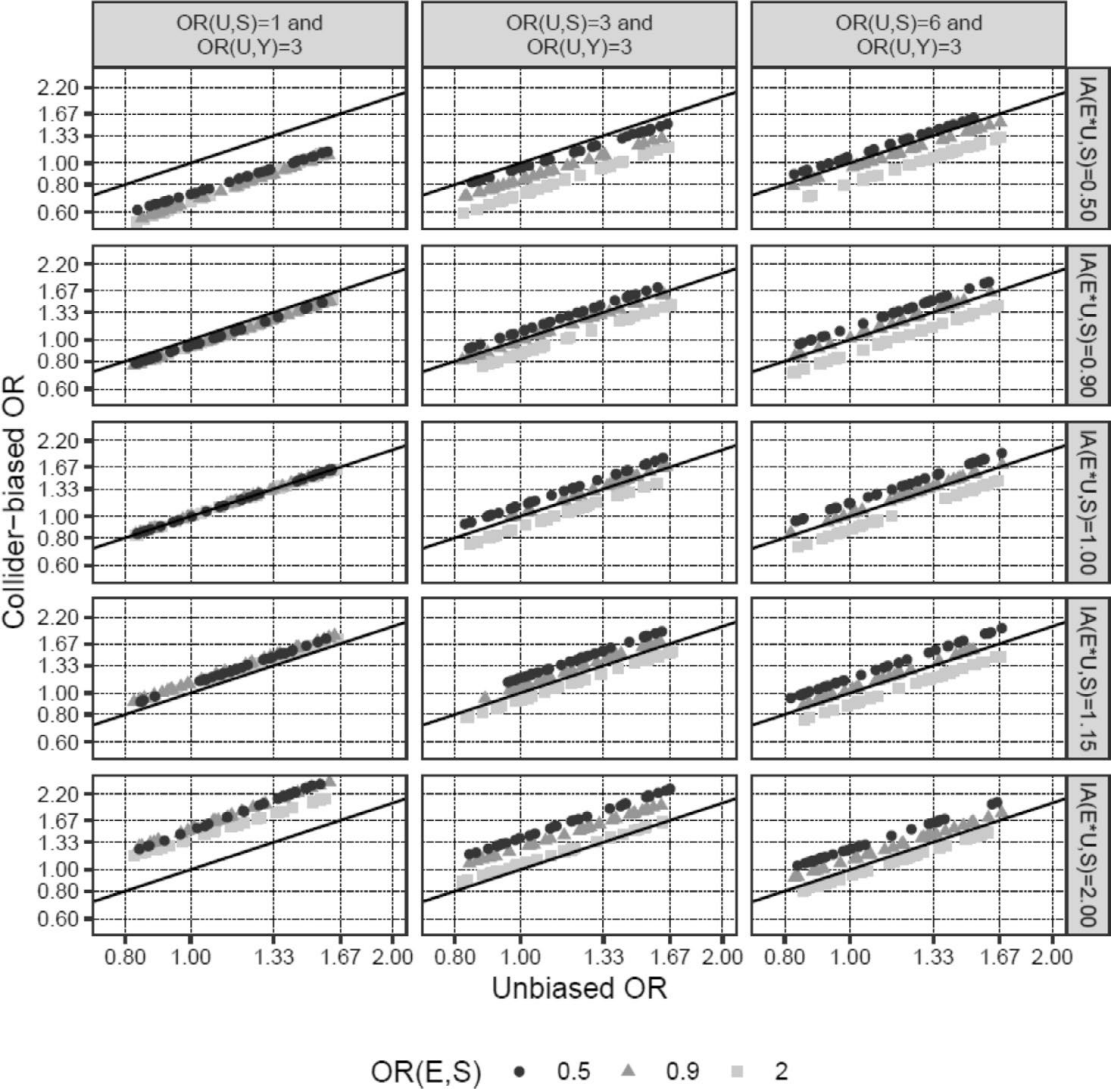
Unbiased (x‐axis) versus collider‐biased (y‐axis) marginal ORs of E on Y in the absence (third row) versus presence (first [OR_IA(E,U)→S_ = 0.5], second [OR_IA(E,U)→S_ = 0.9], fourth [OR_IA(E,U)→S_ = 1.15], and fifth [OR_IA(E,U)→S_ = 2.0] row) of an interaction between E and U on S on the log(OR)‐scale. The three columns show the results for scenarios with different strengths of the association of U with S (OR_U→S_ set to 1 [first column], 3 [second column], and 6 [third column]). The association between U and Y (OR_U→Y_) is fixed at 3, and disease (p_S,BL_) and disease outcome (p_Y,BL_) baseline probabilities are fixed at 0.2 in all scenarios. One dot/mark corresponds to one simulation based on a sample size of 500,000. The third row without interactions is in line with Figure 2. Of note, in the first column of row three, U does not have an association with S at all (both OR_U→S_ and OR_IA(E,U)→S_ are 1), thus no collider bias can be introduced. For an OR_IA(E,U)→S_ of 0.9 and 1.15 (rows two and four) the collider bias is still of relatively moderate size if the OR_U→S_ is 0.9, but might already be relevant for stronger associations (in terms of OR_U→S_) between U and S. For OR_IA(E,U)→S_ values of 0.5 and 2 (rows one and five) the amount of collider bias is substantial, and the biased vs. the unbiased odds ratios are of opposite directions for a large range of OR values. Interestingly, in the case of the presence of strong interaction (rows one and five), the amount of collider bias tends to increase with smaller main effects of U on S (OR_U→S_) (column one vs. two vs. three), at least as long as the main effect of U on E (OR_U→E_) is small (e.g., 0.9), which is not true in the absence of interaction (row three). In summary, the impact of the interrelationship between OR_E→S_, OR_U→S_, and OR_IA(E,U)→S_ on the amount of collider bias appears to be complex and quite unpredictable. However, for the range of input parameters which appear plausible for the PCa scenario, the amount of collider bias is most likely small, even in the case of (not too strong) interactions. OR, odds ratio.

Next, we investigated how changes in the input parameters affect the magnitude of the bias. Increasing the effect of E on S moderately increased the percentage bias (Table 1; scenarios 11 and 12 vs. 1). The interaction between E and U on S was a very potent factor in introducing collider bias (scenarios 13 and 14), even more so when the main effect of U on S was absent (scenario 15 vs. 14), and was able to reverse the direction of the biased versus the unbiased OR in scenarios 14 and 15. In contrast, the probabilities of disease (*p*_S,BL_) and outcome (*p*_Y,BL_), as well as the specific value of the OR of E on Y, only marginally affected the percentage bias (scenarios 16–18 vs. 1, scenario 19 vs. 17). It is noteworthy, however, that bias, even if it is of the same size in terms of percentage bias, impacts the interpretation of results much more for small effect sizes of E on Y (scenario 19, OR_E→Y_ = 1.04), since even a small bias could change a slightly positive to a slightly negative effect (e.g., an OR from 1.04 to 0.99), while the interpretation of large effects is much less affected by such minor changes (e.g., 2.00 to 1.95). Finally, the direction of the bias was determined by the signs of the associations of U with S and Y (scenario 20). These findings are also visualized in Figures 2 and 3. In summary, the E–U interaction on S is the parameter that has the strongest effect on the amount of collider bias, and the impact of the interrelationship between the main effects of E and U on S together with the interaction term on the amount of collider bias appears to be complex and unpredictable.

**FIGURE 4:**
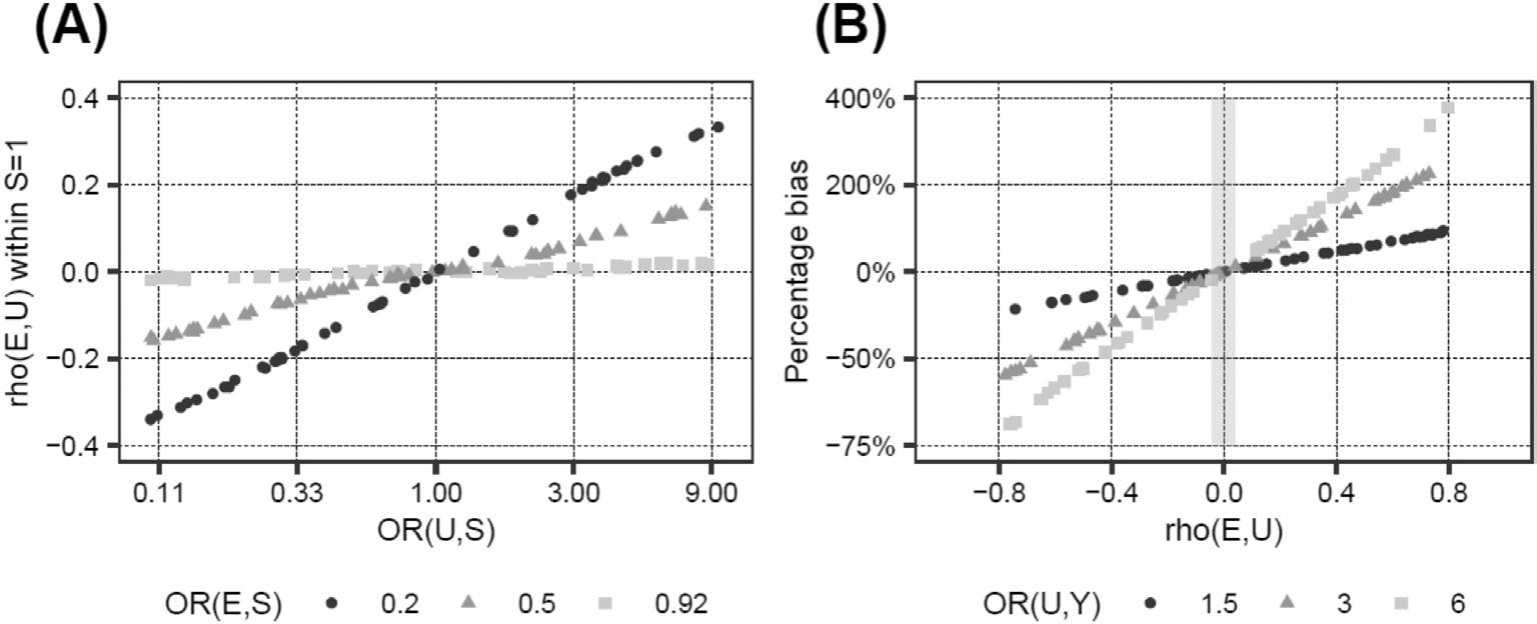
Collider stratification induced correlation between E and U as a function of the associations between E and S as well as U and S in the absence of interaction (A), and the percentage bias as a function of the correlation between E and U and the association between U and Y (B). For panel (A), independent, *N* (0, 1)‐distributed variables E and U, acting on a binary variable S (S = 1 with baseline probability 0.04, S = 0 with baseline probability 0.96) with odds ratios OR_E→S_ and OR_U→S_ and no interaction (OR_IA(E,U)→S_ = 1) were simulated, and Pearson correlation coefficients ρ between E and U within the subset S = 1 were calculated. One dot/mark corresponds to one simulation based on a sample size of 2,500,000. For an OR_E→S_ of 0.92, which is a plausible estimate for the association between BMI and localized PCa, ρ remained very small (i.e., |ρ| < 0.02) even for large values of OR_U→S_. Strong effects of both E and U on S are necessary to obtain values of ρ which are substantially different from 0. For panel (B), E and U following a bivariate standard normal distribution with correlation $\rho \;\left(E,U\right)$ were simulated, acting on a binary variable Y (*Y* = 1 with baseline probability 0.04, Y = 0 with baseline probability 0.96) with odds ratios OR_E→Y_ (fixed at 1.12) and OR_U→Y_ (taking values of 1.5, 3, and 6), and the percentage bias was calculated. The percentage bias was calculated as $\left({\text{OR}}_{\text{Biased}}-{\text{OR}}_{\text{True}}\right)/{\text{OR}}_{\text{True}}\times 100$, where ${\text{OR}}_{\text{Biased}}$ is the unadjusted (“confounder‐biased”), and ${\text{OR}}_{\text{True}}$ the (for U) adjusted (“unbiased”) marginal OR of E on Y. One dot/mark corresponds to one simulation based on a sample size of 500,000. The stronger the correlation ρ between E and U, and the larger OR_U→Y_, the higher the percentage bias. Specifically, ρ values between −0.04 and 0.04 (indicated by the slim gray shaded area) as observed in the simulations mimicking the PCa scenario (also including interaction effects; Table 1, scenarios 1–10), are not sufficient to introduce relevant bias. However, for unmeasured/unknown classical confounders larger ρ values and thus relevant confounder bias are conceivable. OR, odds ratio.

As seen in the last column of Table 1, the collider stratification induced association between U and E was small for all plausible BMI–PCa scenarios (Pearson correlation coefficient ρ < |0.04|), compared to the original association between U and S (OR_U→S_ = 3 in most scenarios, corresponding to ρ~0.2 for *p*_S,BL_ = 0.04). Since for a confounder of the relationship between E and Y the correlation ρ can easily be larger than 0.04, the bias introduced by ignoring such a confounder might be substantially larger than the collider bias introduced by ignoring a confounder of the relationship between S and Y. Indeed, modifying U from an S–Y confounder to a classical E–Y confounder in scenario 5 of Table 1, while leaving all other input parameters unchanged, led to a confounder‐induced percentage bias of 240%, compared to < 2% in the original collider bias setting. Figure 4 illustrates this observation.

## 4 Discussion

Using simulations, we examined the extent to which the observed association between BMI and PCa death in localized PCa cases might be affected by collider stratification bias originating from the analysis of cases only. We specifically scrutinized the results of a recent Swedish cohort study of approximately 370,000 men, where a 5‐kg/m^2^ higher BMI increased the risk of PCa‐specific death in localized PCa cases by 18% (HR = 1.18, 95% confidence interval: 1.01–1.37) [2]. Our simulations showed that in this setting, the influence of collider bias is small and is an insufficient explanation for the inverse obesity paradox in PCa. This finding is robust and holds for plausible variations in the input parameters, as long as we do not assume an unreasonably high interaction between BMI and the unmeasured and/or unknown risk factors for PCa diagnosis. Collider bias distorts HRs (approximated by ORs in our simulations) by no more than 4% on the relative percentage bias scale. This corresponds to a distortion of roughly ±0.04 for the actual HR point estimate if the HR is not too far away from 1, as is the case for continuous BMI. Thus, the random variability in the PCa death HR estimate due to the finite sample size (the 95% confidence interval reported in the Swedish study mentioned above spans a range of 0.36) outweighs the collider bias by far.

As PCa is one of the most heritable cancers [20], genetic risk factors for PCa development and progression are strong candidates for introducing collider bias into PCa case‐only survival analyses. Genome‐wide association studies have identified nearly 300 germline genetic variants associated with PCa risk, as well as PCa mortality [21, 22, 23, 24, 25]. The derived polygenic risk scores (PRS) for PCa are potent predictors of PCa risk, and HRs for PCa risk as high as 11 (comparing the top 20% vs. bottom 20% of the PRS) have been reported [25]. Converting this HR back to the continuous PRS scale (assuming linearity) gives an HR of ~2.4 per 1‐SD increase. HRs reported in most other studies were slightly smaller, usually converting to HRs of ~2.0 per 1‐SD on a continuous scale [21, 22, 23]. However, such PRSs do not capture all genetic risk; some common variants with small effects may still be undetected, and rare variants with large effects are also not included. Indeed, one study demonstrated a substantially higher risk of PCa in carriers of rare, highly penetrant PCa genetic risk variants compared to non‐carriers, for both low and high PRS values of common variants [23]. Considering this and the probable existence of yet unknown genetic factors, together with the fact that, beyond genetics, family history of PCa and/or environmental factors might affect PCa risk and mortality, the choice of ORs of 3 per 1‐SD increase for the U→S and U→Y relationships in most of our simulations in Table 1 seems reasonable. However, we also investigated ORs as high as 10, and even in these extreme scenarios, the magnitude of the collider bias remained small. Notably, strong associations of U with both S and Y are necessary for the induction of collider bias, and only one strong association is not sufficient. Other variables, such as sociodemographic factors, are already routinely adjusted for in observational studies, and can thus be ruled out as drivers of collider bias.

In the investigated scenario of PCa‐specific mortality, the potential for collider bias is small because the negative association of BMI with the likelihood of a diagnosis of localized PCa is small (HR per 5‐kg/m^2^ ~ 0.9). For collider bias to become relevant, a substantially stronger association between BMI and localized PCa diagnosis is required. This conclusion does not depend on the specific cumulative incidence of PCa death (i.e., *p*_Y,BL_), and thus would also hold for associations obtained from studies with a follow‐up time resulting in a different, potentially larger, cumulative incidence than the 4% used in most of our simulations. Modification of the effect of BMI on PCa diagnosis by U is an especially influential and uncertain factor in introducing collider bias [14, 15] (Figure 3). However, we could not find any reports of BMI effect modification by genetic PCa risk in the literature; therefore, our assumption that, if present, the effect size of such an interaction is at most half the size (ORs 0.96 and 1.04) of the main effect of BMI on PCa risk (OR 0.92) appears reasonable.

A discussion of the potential of collider bias versus classical confounding bias and other sources of bias in the context of the association of obesity with PCa‐specific mortality is warranted. Our simulations demonstrated that classical confounding bears a higher potential of relevantly affecting the observed results than collider stratification. Even relatively modest confounding, including residual confounding, e.g., for smoking, might distort the observed associations by a larger margin than that introduced by collider stratification in PCa cases only. However, to completely explain away the HR of ~1.2 for the relationship between BMI and PCa death (i.e., percentage bias ≥ 20%), very strong confounding would be necessary (Figure 4B); the existence of such confounders beyond the variables already routinely adjusted for in contemporary observational studies [2, 3] is very unlikely. Furthermore, full population and case‐only analyses should be affected similarly, and thus classical confounding is also not an explanation for the often observed obesity paradox. Detection bias is another type of bias with a higher potential to distort observed associations than collider bias [4, 26, 27]. Detection bias is quite plausible in full population analyses considering that delayed detection of PCa is more likely in men with obesity compared to normal weight men because of hemodiluted PSA levels, enlarged prostate glands, and possibly lower frequency of asymptomatic PSA screening in men with obesity [2, 4, 26]. By adjusting for clinical characteristics at PCa diagnosis (e.g., TNM staging, PSA level, and Gleason score) in case‐only analyses, as done in our previous study from which we derived effect sizes of BMI on PCa [2], the potential of detection bias was minimized. Moreover, interpreting associations between BMI and PCa mortality might also be complicated by analytical challenges arising from differences in age at PCa diagnosis and differential risks of non‐PCa death according to BMI status, although modern statistical approaches aim to minimize this source of distortion. Taken together, these findings add further evidence to the rationale that the observed positive relationship between BMI and PCa death is real, and not a statistical artifact. Insulin resistance has recently been shown to be an important pathway through which obesity accelerates PCa death in PCa cases, thus providing a biological explanation for the relationship between BMI and PCa death [28]. Other plausible explanations include [4]: (i) alterations in sex hormone metabolism, particularly androgen deficiency [29], (ii) chronic inflammation characterized by altered levels of adipokines in men with obesity [30], and (iii) less successful treatment, such as higher rates of positive surgical margins, in men with obesity, with associated higher rates of disease recurrence [31].

Our simulation approach is flexible and allows the exploration of a wide range of scenarios, including interactions (Figures 2 and 3). However, we are only simulating simple scenarios with one unobserved S‐Y confounder at a time. Furthermore, for our simulations, we used logistic regression models with a binary outcome variable Y, whereas the studies that we were emulating were prospective cohort studies, modeling the time to PCa death using Cox models. However, this simplification (ORs instead of HRs) is often done in simulation analyses and should not substantially affect our findings.

## 5 Conclusions

The results of our simulations demonstrate that collider stratification bias is unlikely to relevantly affect the positive association between BMI and PCa‐specific mortality, as observed in the analyses of localized PCa cases only. Assuming that confounder and detection bias are also unlikely to completely explain away the observed associations due to extensive adjustments of the statistical models adds further evidence to the rationale that the observed positive relationship between BMI and PCa death is real, and not a statistical artifact. Our findings emphasize the importance of exploring alternative mechanisms beyond collider bias to better understand the underlying factors driving this paradox.

## Author Contributions

T.S. was involved in conceptualization, funding acquisition, investigation, and writing – review and editing; C.H.: methodology, validation, and writing – review and editing. J.F. was involved conceptualization, formal analysis, investigation, methodology, visualization, and writing – original draft.

## Ethics Statement

The authors have nothing to report.

## Consent

The authors have nothing to report.

## Conflicts of Interest

The authors declare no conflicts of interest.

## Supporting information


Appendix S1.

## Data Availability

Only simulated data were used. The procedure for generating data is described in the Methods section.
